# StudyTypeTeller—Large language models to automatically classify research study types for systematic reviews

**DOI:** 10.1017/rsm.2025.10031

**Published:** 2025-09-11

**Authors:** Simona Emilova Doneva, Shirin de Viragh, Hanna Hubarava, Stefan Schandelmaier, Matthias Briel, Benjamin Victor Ineichen

**Affiliations:** 1 Center for Reproducible Science, University of Zurich, Zurich, Switzerland; 2 CLEAR Methods Center, Division of Clinical Epidemiology, Department of Clinical Research, University Hospital Basel, University of Basel, Basel, Switzerland; 3 MTA–PTE Lendület “Momentum” Evidence in Medicine Research Group, Medical School, University of Pécs, Pécs, Hungary; 4 Department of Health Research Methods, Evidence, and Impact, McMaster University, Hamilton, ON, Canada; 5 Department of Clinical Research, University of Bern, Bern, Switzerland

**Keywords:** animal study, clinical study, language models, natural language processing, randomized controlled trial, systematic review

## Abstract

Abstract screening, a labor-intensive aspect of systematic review, is increasingly challenging due to the rising volume of scientific publications. Recent advances suggest that generative large language models like generative pre-trained transformer (GPT) could aid this process by classifying references into study types such as randomized-controlled trials (RCTs) or animal studies prior to abstract screening. However, it is unknown how these GPT models perform in classifying such scientific study types in the biomedical field. Additionally, their performance has not been directly compared with earlier transformer-based models like bidirectional encoder representations from transformers (BERT). To address this, we developed a human-annotated corpus of 2,645 PubMed titles and abstracts, annotated for 14 study types, including different types of RCTs and animal studies, systematic reviews, study protocols, case reports, as well as in vitro studies. Using this corpus, we compared the performance of GPT-3.5 and GPT-4 in automatically classifying these study types against established BERT models. Our results show that fine-tuned pretrained BERT models consistently outperformed GPT models, achieving F1-scores above 0.8, compared to approximately 0.6 for GPT models. Advanced prompting strategies did not substantially boost GPT performance. In conclusion, these findings highlight that, even though GPT models benefit from advanced capabilities and extensive training data, their performance in niche tasks like scientific multi-class study classification is inferior to smaller fine-tuned models. Nevertheless, the use of automated methods remains promising for reducing the volume of records, making the screening of large reference libraries more feasible. Our corpus is openly available and can be used to harness other natural language processing (NLP) approaches.

## Highlights

### What is already known?

Screening abstracts for systematic reviews is a labor-intensive process. Large language models, like generative pre-trained transformer (GPT), have been suggested as tools to help categorize abstracts into study types—such as randomized controlled trials (RCTs) or animal studies—before screening, potentially reducing the number of references to review and making the process more efficient. However, there has been no direct comparison of how well GPT performs this task compared to more established models like bidirectional encoder representations from transformer (BERT).

### What is new?

We introduce a new dataset of abstracts, labeled by humans according to 14 different study types. Using this dataset, we evaluated how well different GPT and BERT models classify these study types. Our results show that BERT models are particularly effective at this task, making them a good choice for sorting abstracts before systematic review. In fact, BERT models consistently outperformed the newer GPT models in this specific task.

### Potential impact for RSM readers

Using BERT models to pre-screen abstracts could reduce the workload of systematic reviews, especially for larger collections of references. And importantly, while GPT models have advanced features and are trained on massive datasets, they were consistently outperformed by the more traditional BERT models for this type of classification.

## Abbreviations

**Table:** 

IAA	inter-annotator-agreement
NLP	natural language processing
EBM	evidence-based medicine
ML	machine learning
AUC	area under the receiver operating characteristic curve
CC	contextual clues
ICL	in-context learning
CoT	chain-of-thought
BERT	bidirectional encoder representations from transformers
GPT	generative pre-trained transformer

## 1 Introduction

Systematic reviews are fundamental to evidence-based medicine and research.^1^^,^ ^2^ Yet conducting these reviews is highly time-consuming, especially the screening of abstracts identified during a comprehensive literature search.^3^^,^ ^4^ Typically, this screening requires substantial resources with two independent human reviewers and potentially a third one for resolution. As a result, the manual screening of a large number of abstracts can take months,^5^ a challenge exacerbated by the ever-increasing volume of scientific publications.^6^ One approach to mitigate the screening burden is to use medical subject headings (MeSH), which provide a standardized vocabulary that refines searches and can reduce irrelevant results. However, their coverage can be incomplete or delayed, and exploiting the full potential of MeSH terminology is an area of active research.^7^^,^ ^8^

With the development of natural language processing (NLP) technologies, particularly large language models (LLMs), there is potential to reduce the manual effort involved in abstract screening. A promising approach is to use such models to trim the number of citations before title and abstract screening, e.g., by classifying articles by study types, such as RCTs, animal studies, systematic reviews, or narrative reviews. Traditional machine learning methods, such as support vector machines and convolutional neural networks, have been successfully used to classify RCTs,^9^^,^ ^10^ often outperforming built-in search filters in databases. These models have also been used to develop Multi-Tagger, a system of publication type and study design taggers supporting biomedical indexing and evidence-based medicine. Multi-Tagger covers 50 publication types, including reviews, editorials, and cohort studies.^11^ The release of the transformer architecture in 2017 created a new wave of research into Deep Learning, leading to the appearance of the first LLMs.^12^ It was closely followed by the release of the BERT models, which have since been actively applied to the task of text classification in general and the classification of study types, e.g., in relation to animal-alternative methods.^13^ More recently, generative models, such as generative pre-trained transformer (GPT) and LLaMA, have also been put to the task of text classification.^14^^,^ ^15^ With this, LLMs have proven to reduce the screening burden of large abstract collections.^16^^,^ ^17^

However, key gaps remain: First, current methods lack detailed classification levels for study types needed for specific systematic reviews.^16^ For example, no existing model can automatically classify key study types, such as in vitro studies, animal studies, or studies assessing therapeutic interventions important, for example, for animal or translational systematic reviews.^16^^,^ ^18^^,^ ^19^ Second, despite the growing popularity of generative models like GPT, there is a lack of empirical data on how these newer models perform in text classification against established transformer-based models, such as BERT. Third, a challenge in advancing these technologies is the need for high-quality, manually annotated data, which is essential for fine-tuning and evaluating models’ performance. Without available annotated datasets, the refinement and successful deployment of NLP models in specialized domains remain constrained.^20^

To address these gaps, our study has three objectives: first, to develop a manually annotated dataset of abstracts for various study types found in PubMed; second, use this corpus to train different transformer-based models from the BERT family to automatically classify PubMed abstracts by study type; and third, to compare the performance of these NLP methods with the newer generative LLMs, concretely GPT-3.5 and GPT-4.

## 2 Materials and methods

### 2.1 Approach and study design

We focused our study on neuroscience, one of the largest biomedical research fields. The data used for classification includes titles, abstracts, keywords, and journal names of research studies found on PubMed. We did not conduct a sample size calculation but relied on a convenience sample of slightly over 2,500 abstracts for our human-annotated corpus of 14 study types. This manually annotated corpus was then used to: 1) assess the performance of GPT-based models in automatically classifying these study types and 2) fine-tune various BERT-based models to compare their performance against GPT in classifying these study types. Details are described in the following paragraphs.

### 2.2 Data

#### 2.2.1 Data collection

To obtain the initial set of relevant PubMed-IDs (PMIDs), we searched PubMed using the following search string: “Central nervous system diseases [MeSH] OR Mental Disorders Psychiatric illness [MeSH]” (search date: November 27, 2023). Out of 2,788,345 PMIDs, we randomly sampled 2,000 PMIDs for which we fetched the meta-data (title, abstract, author keywords, MeSH terms, journal name, PubMed date of publication, and date of journal publication). Although author keywords and MeSH terms may appear similar, they serve different purposes: Authors typically choose a small set of keywords based on the focus of their article, while indexers systematically assign standardized MeSH terms to place the work within a broader framework.^21^

#### 2.2.2 Data annotation

We developed detailed annotation guidelines for the different study types which were iteratively improved through three rounds of revision (see Section a of the Supplementary Material or https://osf.io/3yxqh/). The final guidelines are provided in Table S1 in the Supplementary Material. We use a classification system that categorizes study types according to their methodological design, study population, and intervention type. We defined “intervention” specifically in the context of therapeutic intervention, excluding diagnostic procedures or similar procedures. We pre-defined the following 15 study type classes, which are largely mutually exclusive and applied in the following hierarchy:

1. *Study-protocol*
2. *Human-systematic-review*
3. *Non-systematic-review*
4. *Human-RCT-non-drug-intervention* (RCT)
5. *Human-RCT-drug-intervention*
6. *Human-RCT-non-intervention*
7. *Human-case-report*
8. *Human-non-RCT-non-drug-intervention*
9. *Human-non-RCT-drug-intervention*
10. *Animal-systematic-review*
11. *Animal-non-drug-intervention*
12. *Animal-drug-intervention*
13. *Animal-other*
14. *In-vitro-study*
15. *Remaining* (a class for all remaining study types not belonging to any of the above mentioned classes).

Notably, we made a post-hoc decision to exclude the class *Animal-systematic-review* as we did not identify any such studies in our corpus (see Section 2.2.3). This left us with 14 classes. We will refer to this number of classes in the following.

Two raters (SdV and BVI) independently assigned these study type classes to the set of 2000 references based on title, abstract, and journal name. Conflicts were resolved by discussion. To assess inter-rater agreement, we calculated Cohen’s Kappa statistics. Four references were excluded in accordance with the annotation guidelines (not being in the realm of neuroscience and/or not in English). All annotations and the conflict resolution were performed in the web-based annotation tool Prodigy.^22^

Next, to create labels for the binary classification task, we mapped each of the classes either to “Animal” or “Other.” This allowed us to evaluate the models’ abilities in distinguishing between animal and non-animal studies.

#### 2.2.3 Dataset enrichment

The manually annotated corpus showed a very uneven distribution of study types, with some classes having very few samples (e.g., *Human-RCT-non-intervention*) and the *Remaining* class containing three times as many samples as the second largest class. Fine-tuning BERT-based models on this unbalanced dataset poses a risk that the model will be biased towards over-predicting the larger classes and performing poorly on the underrepresented classes. See Figure 6a in the Supplementary Material and Section g of the Supplementary Material for the effect of enrichment on the performance of BERT-based models. At the same time, having very few examples of a class in our test set can lead to unreliable performance metrics, high variance in evaluation results, and poor representation of real-world performance. We thus made a post hoc decision to augment each of the minority classes with 50 additional abstracts (Table 1). These additional instances stemmed from previous systematic reviews conducted by our group.

**Table 1:** Annotated corpus: The table presents key statistics of the dataset after the stratified splitting into train, validation, and test sets

	Train	Validation	Test	**Total**
**Corpus size**	1,581	530	534	**2,645**
**Binary labels**
Other	1,354	455	457	**2,266**
Animal	227	75	77	**379**
**Multiclass labels**
Remaining	514	172	172	**858**
Non-systematic-review	222	74	75	**371**
Human-non-RCT-non-drug-intervention	123	41	42	**206**
Human-non-RCT-drug-intervention	107	36	36	**179**
Human-case-report	99	33	33	**165**
Animal-other	93	31	31	**155**
Animal-drug-intervention	88	29	30	**147**
Human-systematic-review	66	22	22	**110**
In-vitro-study	62	21	21	**104**
Human-RCT-non-drug-intervention	52	18	18	**88**
Animal-non-drug-intervention	46	15	16	**77**
Human-RCT-drug-intervention	43	15	15	**73**
Clinical-study-protocol	34	12	12	**58**
Human-RCT-non-intervention	32	11	11	**54**

*Note*: The numeric values represent the number of records in the respective category

#### 2.2.4 Data splits for model training and evaluation

We split the full dataset of 2,645 references into three datasets for training, validation, and testing with a 0.6-0.2-0.2 ratio, resulting in sub-corpora of 1,851, 530, and 534 samples, respectively (Table 1). The train and validation split are only used for fine-tuning the BERT-based models, while the held-out test set is used for both BERT and GPT evaluation. A random sample from the validation dataset was used to select the most promising GPT prompts (see Section 2.3.2).

To ensure that all classes are present across all parts of the split, we employed a stratification strategy which ensured that all classes were present across all dataset splits. Each dataset record included the following fields: title, abstract, author keywords, and journal name. Note that we did not include MeSH terms, as empirical evaluations for a similar task have shown no positive impact on performance.^13^

### 2.3 GPT-based models

#### 2.3.1 Setup

We employed two generative LLMs developed by OpenAI: GPT-3.5-turbo and GPT-4-turbo-preview.^23^^,^ ^24^ These models have been trained on publicly available data (e.g., Common Crawl, a public dataset of web page data [https://commoncrawl.org] and Wikipedia) and licensed third-party data.^23^^,^ ^24^ GPT models generate text by predicting the next word in a sequence, based on the context of the previous words. GPT-4 is an advancement over GPT-3.5, featuring a larger model size, enhanced performance (including in the domain of medicine),^25^^,^ ^26^ and more extensive training data.

We utilized the OpenAI chat-completion API to obtain responses from both models.^27^ Each abstract’s classification was run in an independent session, and since the API currently does not have a memory function, there is no transfer of information or bias from one abstract to another. The classification task was structured as a question-answering problem, where GPT was prompted to identify the most suitable study type from a predefined study type set based on the paper’s title, abstract, journal name and, when available, keywords. The analysis was conducted with the temperature parameter set at 0 to reduce the randomness of responses (compared to higher values producing more creative and random outputs).

Furthermore, to deal with variation in GPT responses, we configured the output format of the API call to a specific structured output type (“json_object”). Additionally, we used a fuzzy matching approach to account for minor differences between model outputs and expected labels.

#### 2.3.2 Prompting strategy

We pre-defined three prompting strategies:

1. **Zero-shot^28^^,^ ^29^**
  The model is given the classification prompt without any additional context, examples, or specific instructions, relying solely on its pre-trained knowledge.
2. **contextual clues (CC)^30^**
  The model is provided with our detailed annotation guidelines (see Section a of the Supplementary Material and Table S1 in the Supplementary Material for the full version and Section b of the Supplementary Material and Table S2 in the Supplementary Material for the shortened version). These guidelines outline the classification criteria and labeling conventions, enabling the model to use these information to create an output.
3. **chain-of-thought (CoT)^30^^,^ ^31^**
  The model is instructed to generate a chain of thought (step-by-step reasoning) before providing its final classification. This process encourages explicit reasoning and can improve accuracy by making the decision process more transparent.

For prompt engineering, i.e., designing the input instructions given to the model, we followed OpenAI’s Prompt Engineering Guidelines^30^ and other published recommendations.^32^ Prompts are summarized in Table 2 and detailed in Section j of the Supplementary Material.

**Table 2:** Overview of employed prompting strategies

Prompt	Prompting	Prompt description
number	strategy	
P1	zero shot	“Classify this text to one of these labels.”
P2	zero shot	“Determine which of these labels fits the text best.”
P2_1	zero shot	added “The classification applies to the journal name, title, and/or abstract of a study.”; output in subsample wrong therefore didn’t run over test data
P3	CC	shortened annotation guidelines, only table; output in subsample wrong therefore didn’t run over test data
P3_1	CC	shortened annotation guidelines, only table; added separate mention of labels; reduced mentions of 1) what classification should apply to and 2) format and changed where those informations occur
P3_2	CC	see above
P3_3	CC	see above
P3_4	CC	shortened annotation guidelines, only table; based on P3_2, changed wording: “list of annotations guidelines” to “following information for classification”
P4	CC	shortened annotation guidelines; output in subsample wrong therefore didn’t run over test data
P4_1	CC	shortened annotation guidelines, based on P3_2 (better wording than P4 and most successful of P3-strategies)
P4_2	CC	shortened annotation guidelines, based on P4_1 but “clinical study protocol” in guidelines instead of “study protocol” (P4_2)
P5	CC	original annotation guidelines, only table
P6	CC	original annotation guidelines
P7	CoT	P1 + short explanation why label was chosen
P8	CoT	step-by-step labeling; didn’t work because output format mostly wrong; for working prompt, see hierarchical labeling
P9	CoT + CC	short explanation why label was chosen + shortened annotation guidelines, only table
P9_1	CoT + CC	same as P9 but with CoT-wording from P7
P10	CoT	“Let’s think step by step.”
P11	CoT + CC	“Let’s think step by step.” + shortened annotation guidelines, only table
P11_1	CoT + CC	“Let’s think step by step.” put in different place than P11 + shortened annotation guidelines, only table
P11_2	CoT + CC	“Let’s think step by step.” put in different place than P11 and P11_1 + shortened annotation guidelines, only table
P11_3	CoT + CC	based on P11 (CoT placement with best results) but with original annotation guidelines, only table
P11_4	CoT + CC	based on P11 but with original annotation guidelines
P11_5	CoT + CC	based on P11 but with shortened annotation guidelines
P12	2 CoT + CC	P11 + added explanation (second CoT) based on P9
P12_1	2 CoT + CC	P11_3 (second-best performance of P11s) + added explanation (second CoT) based on P9
P12_2	2 CoT + CC	P11_4 (best performance of P11s) + added explanation (second CoT) based on P9

*Note*: Detailed prompts are listed in Section j of the Supplementary Material.

The “original annotation guidelines” refer to the complete guidelines (see Section a of the Supplementary Material and Table S1 in the Supplementary Material).

For the annotation guidelines, see Sections a and b of the Supplementary Material and Tables S1 and S2 in the Supplementary Material.

Abbreviations: CC, contextual clues; CoT, chain-of-thought.

We tested both, 1) classification into one of 14 categories (multi-class) or 2) just two (“Animal” versus “Other,” binary) due to the following reasons: First, it allowed us to compare the performance of BERT-based and GPT-based models on a simpler binary classification task. Second, it allowed us to construct a hierarchical classification pipeline, i.e., a two-step approach where binary classification is followed by more detailed classification (i.e., a form of the Chain-of-Thought-prompting-strategy). We also briefly explored in-context learning (few-shot)^31^ as a prompting strategy, i.e., giving the model a few examples in the prompt (Table S10 in the Supplementary Material). The selection of few-shot examples was based on,^33^^,^ ^34^ as follows:

1. **Random Few-Shots**: In this approach, the classification examples (few-shots) were randomly selected from the training split.
2. **K-Nearest Neighbor (K-NN)**: Here, the few-shot examples were chosen based on the closest neighbors to each test example from the training split.

#### 2.3.3 Experiments

We first tested the prompting strategies on random samples of 50 abstracts from the validation dataset. To compare GPT with BERT performance, we re-ran all the prompts on the test set (enriched with keywords), see Table S8 in the Supplementary Material. In addition, we tested them on the test split without keywords (Table S9 in the Supplementary Material).

Due to relatively high API costs of GPT-4, we decided to only run the most promising prompts (based on their performance with GPT-3.5) and at least one prompt of each prompting strategy on GPT-4. In total, we tested eight selected prompts on GPT-4 (Table 3).

**Table 3:** Performance metrics for GPT-3.5-turbo and GPT-4-turbo-preview (for selected prompting strategy) as well as for BERT models (multi-class classification)

Prompt	Concept	Precision (CI)	Recall (CI)	F1-Score (CI)
GPT-3.5
P1	zero-shot	0.454 (0.351, 0.539)	0.331 (0.292, 0.371)	0.261 (0.22, 0.305)
P4_1	CC	0.563 (0.498, 0.611)	0.434 (0.393, 0.476)	0.416 (0.372, 0.461)
P5	CC	0.608 (0.54, 0.65)	0.511 (0.466, 0.552)	0.498 (0.452, 0.542)
**P6**	**CC**	**0.591 (0.536, 0.631)**	**0.541 (0.5, 0.584)**	**0.532 (0.487, 0.575)**
P7	CoT	0.467 (0.334, 0.589)	0.305 (0.266, 0.345)	0.229 (0.19, 0.269)
P11_3	CoT + CC	0.636 (0.577, 0.685)	0.504 (0.461, 0.545)	0.488 (0.444, 0.534)
P11_4	CoT + CC	0.601 (0.543, 0.644)	0.528 (0.487, 0.571)	0.518 (0.474, 0.564)
P12_2	2 CoT + CC	0.605 (0.548, 0.651)	0.464 (0.425, 0.507)	0.445 (0.398, 0.491)
**P2_H**	**b_P3, CC**	**0.617 (0.561, 0.655)**	**0.551 (0.507, 0.592)**	**0.54 (0.495, 0.583)**

GPT-4			
P1	zero-shot	0.59 (0.519, 0.651)	0.472 (0.429, 0.515)	0.428 (0.382, 0.474)
P4_1	CC	0.638 (0.584, 0.684)	0.556 (0.513, 0.597)	0.534 (0.488, 0.578)
P5	CC	0.701 (0.652, 0.738)	0.592 (0.551, 0.633)	0.588 (0.546, 0.63)
**P6**	**CC**	**0.709 (0.665, 0.744)**	**0.603 (0.562, 0.644)**	**0.600 (0.555, 0.642)**
P7	CoT	0.584 (0.485, 0.657)	0.459 (0.418, 0.502)	0.402 (0.355, 0.447)
P11_3	CoT + CC	0.696 (0.646, 0.736)	0.588 (0.547, 0.631)	0.582 (0.536, 0.624)
P11_4	CoT + CC	0.703 (0.658, 0.74)	0.601 (0.56, 0.642)	0.596 (0.552, 0.638)
P12_2	2 CoT + CC	0.682 (0.631, 0.72)	0.569 (0.526, 0.61)	0.563 (0.519, 0.606)
**P2_H**	**b_P3, CC**	**0.741 (0.699, 0.772)**	**0.644 (0.603, 0.684)**	**0.645 (0.603, 0.686)**

BERT models			
Bio_ClinicalBERT		0.802 (0.762, 0.832)	0.803 (0.768, 0.835)	0.801 (0.764, 0.835)
BERT-base		0.820 (0.781, 0.848)	0.820 (0.785, 0.852)	0.815 (0.777, 0.848)
BioBERT		0.827 (0.789, 0.855)	0.828 (0.794, 0.858)	0.824 (0.788, 0.855)
PubMedBERT		0.826 (0.786, 0.853)	0.828 (0.794, 0.858)	0.824 (0.790, 0.856)
BiomedBERT		0.846 (0.808, 0.872)	0.841 (0.809, 0.871)	0.838 (0.804, 0.868)
BioLinkBERT		0.842 (0.803, 0.867)	0.841 (0.807, 0.869)	0.839 (0.805, 0.869)
**SciBERT**		**0.843 (0.805, 0.869)**	**0.839 (0.805, 0.869)**	**0.839 (0.806, 0.869)**

*Note*: Text in bold indicates the best performance

Performance of GPT models is measured on the enriched dataset with keywords.

Abbreviations: CC, contextual clues; CoT, chain-of-thought; “P2_H,” “P2_HIERARCHY,” i.e., hierarchical prompt 2; “b_P3,” binary prompt 3, upon whose outputs this hierarchical labeling was based (see Tables S6 and S7 in the Supplementary Material).

### 2.4 BERT-based models

#### 2.4.1 Setup

BERT differs from GPT primarily in its training objective. While GPT is trained to predict the next token in a sequence using only the left (previous) context in a unidirectional manner, BERT uses a bidirectional approach through masked language modeling (MLM).^35^ In MLM, random tokens are masked during training, and BERT learns to predict them using both the left and right context. This bidirectional conditioning enables BERT to capture richer contextual representations.^35^^,^ ^36^

We fine-tuned seven BERT models (accessed via https://huggingface.co/):

- bert-base-uncased (BERT-base);^35^
- scibert_scivocab_uncased (SciBERT);^37^
- biobert-v1.1 (BioBERT);^38^
- Bio_ClinicalBERT (ClinicalBERT);^39^
- BiomedNLP-BiomedBERT-base-uncased-abstract (BiomedBERT);^40^
- BiomedNLP-PubMedBERT-base-uncased-abstract-fulltext (PubMedBERT);^41^
- BioLinkBERT-base (BioLinkBERT).^42^

Hyperparameter optimization is detailed in Table S4 in the Supplementary Material and Section e of the Supplementary Material. For more detailed information about the models, see Section d of the Supplementary Material. Model selection was guided by the considerations of accessibility, domain-specificity, performance, but also size, giving preference to smaller (base) versions of the models. We also took into account the BLURB leaderboard of models in the domain of biomedicine.^41^

The input text consisted of the journal name, title, abstract, and—if available—keywords, combined into a single string. We did not apply further text preprocessing. Instead, we used the built-in tokenizers (AutoTokenizers) provided with each model to convert the text into tokens. Inputs were padded or cut off to a maximum length of 256 tokens (approximately 200–250 words), based on prior hyperparameter tuning. Default model settings and hyperparameters were used unless specified otherwise in Table S4 in the Supplementary Material and Section e of the Supplementary Material.

We fine-tuned on the train split and validated on the val (validation) split. The fine-tuned models were then evaluated on the previously unseen held-out test split.

#### 2.4.2 Experiments

For all experiments with BERT-based models, we used two classification types: binary and multi-class (see Section 2.3.2).

### 2.5 Comparative analysis with existing datasets

As a simple baseline for the binary classification task, we used the MeSH terms assigned to the studies. If they contained “animal,” the study was assigned this label, while the remaining studies were assigned the label “other.”

To validate our dataset, we compared it with two recently published abstract classification corpora, *Multi-Tagger* and *GoldHamster*.^11^^,^ ^13^^,^ ^43^ The *GoldHamster* dataset supports classification of PubMed abstracts into experimental models, including “in vivo,” “organs,” “primary cells,” “immortal cell lines,” “invertebrates,” “humans,” “in silico,” and “other.” Due to incomplete documentation, we re-implemented their model locally and fine-tuned it on the provided dataset before applying it to the 2,645 abstracts in our corpus. A direct performance comparison with *GoldHamster* was only feasible for binary classification, where we mapped the “in_vivo” label to “Animal” and all others to “Other.” The additional categories were analyzed to gain further insight into our annotations.

*Multi-Tagger* focuses on human-related studies, providing classification for 50 labels on publication types (e.g., review and case report) and clinically relevant study designs (e.g., case-control study and retrospective study). We retrieved the predicted probability scores for all PubMed articles (up to 2024) from the publicly available dataset at Multi-Tagger File Download, along with threshold values reported to yield the highest F1-scores. Although these labels were not directly comparable to our own, they offered additional context and aided our understanding of the scope and consistency of our annotations.

### 2.6 Performance evaluation

To evaluate classification performance in our text classification task, we treat each label in a one-versus-rest manner, thus transforming the problem into a set of binary classification tasks. This allows us to assess how well the model identifies each individual class against all others.

For each class, we compute precision, recall, and F1 score, defined as:

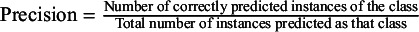
 , also referred to as positive predictive value.

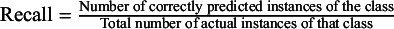
 , also referred to as sensitivity.

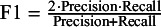
 , i.e., the harmonic mean of precision and recall.

To assess the reliability of these metrics, we compute confidence intervals. Precision and recall intervals are calculated using *binomial proportion methods*,^44^ while F1 confidence intervals are estimated analytically following.^45^

For aggregated (multi-class) performance, we report weighted precision, recall, and F1 scores. These are computed as class-wise scores weighted by the number of true instances in each class: 

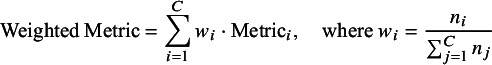

with *n*_*i*_ being the number of true instances in class *i*, and *C* the number of classes. This formulation accounts for class imbalance.

Confidence intervals for these aggregated metrics are computed using bootstrapping, where we resample the test set (with replacement) multiple times, compute the metrics for each sample, and derive empirical confidence intervals from the resulting distributions. All calculations are based on validated implementations from a publicly available Python library.^46^ More mathematical detail is provided in Section c of the Supplementary Material.

## 3 Results

### 3.1 Corpus

**Figure 1:**
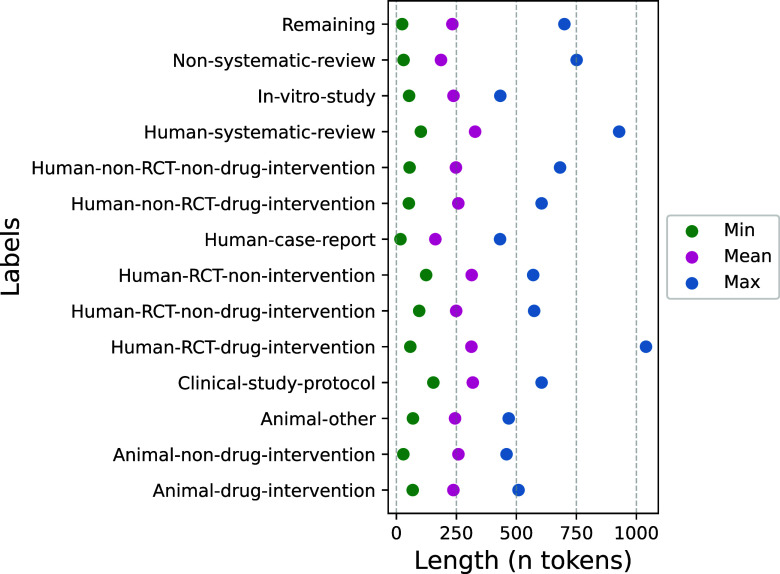
Abstract length per class, calculated on the whole dataset before splitting into train, validation, and test sets.

The final corpus contained 2,645 samples (1,996 from the original annotation and 649 added to increase underrepresented classes) (Table 1). We encountered no error messages from the PubMed API and successfully retrieved all available data. All records included a title and abstract, while 6 lacked a journal name and 18 were missing MeSH terms. The largest proportion of missing data was for author keywords, which were absent in around 60% of records across all labels, except for Human-Systematic-Review and Clinical-Study-Protocol, which had about 30% missing (Figure 5 in the Supplementary Material). The inter-annotator-agreement (IAA) for the human annotation according to Cohen’s Kappa was 0.55 (95%-CI: 0.49–0.61, moderate agreement) for the first round and 0.71 (0.63–0.79, substantial agreement) for the second round. The most represented class was the *Remaining* class with a total of 858 samples, followed by *Non-systematic-review* with 371 samples. The smallest class was *Human-RCT-non-intervention* with 54 samples. 379 samples were animal studies and 2,266 were non-animal studies. The mean abstract length was 237 words (min 17–max 1040) (Figure 1 and Table S3 in the Supplementary Material).

### 3.2 GPT models

**Figure 2:**
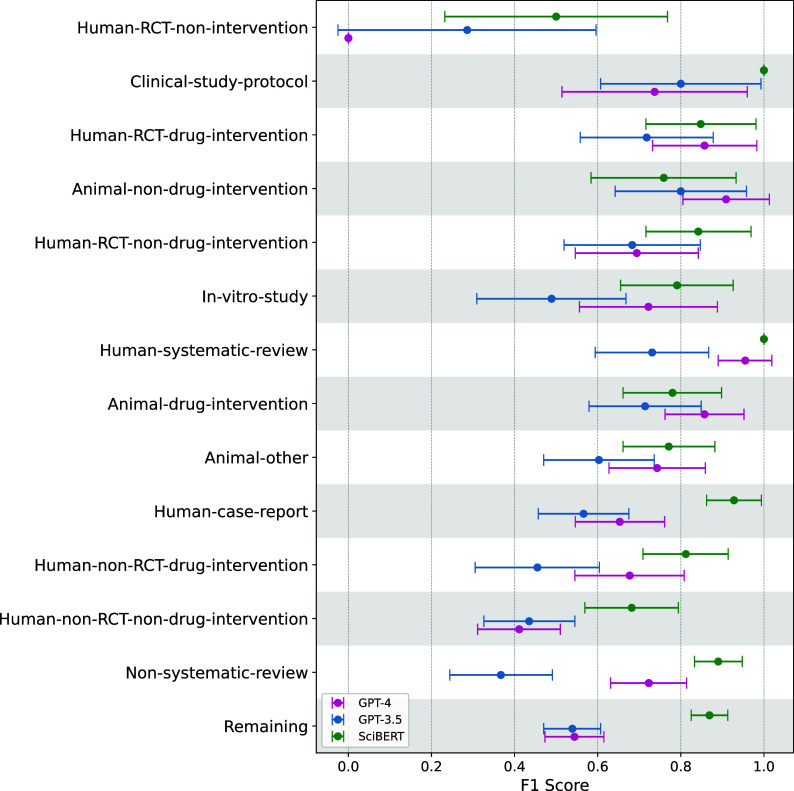
Per-class performance comparison between the best performing prompting strategy for GPT-3.5 and GPT-4 (P2_H_b3, CC), and SciBERT with 95%-confidence intervals.

GPT models performed almost perfectly in the binary text classification task (i.e., “Animal” versus “Other”) with F1-scores up to 0.99 (Table S6 in the Supplementary Material). However, this performance dropped substantially for multi-class classification (considering all 14 study classes) with F1-scores between 0.261 and 0.645. GPT-4 consistently outperformed GPT-3.5 (maximum F1-scores 0.540 versus 0.645, respectively) (Table 3 and Figure 2) (only the most promising prompts were tested on GPT-4).

For multi-class classification, the best performing prompting strategy was hierarchical prompting, i.e., to first classify into “Animal” studies versus “Other” (non-animal) studies, followed by further sub-classification into the 14 classes (with the highest performing prompt being “P2_H_b3” “P2_HIERARCHY” based upon binary prompt P3, Table S7 in the Supplementary Material). The second-best performing prompt was multi-class-prompt P6 (adding the complete annotation guidelines to the GPT-prompt). The lowest performing strategy was zero-shot prompting, i.e., prompting the model without any additional context. All performance metrics for GPT-3.5 are reported in Tables S7 and S8 in the Supplementary Material.

**Figure 3:**
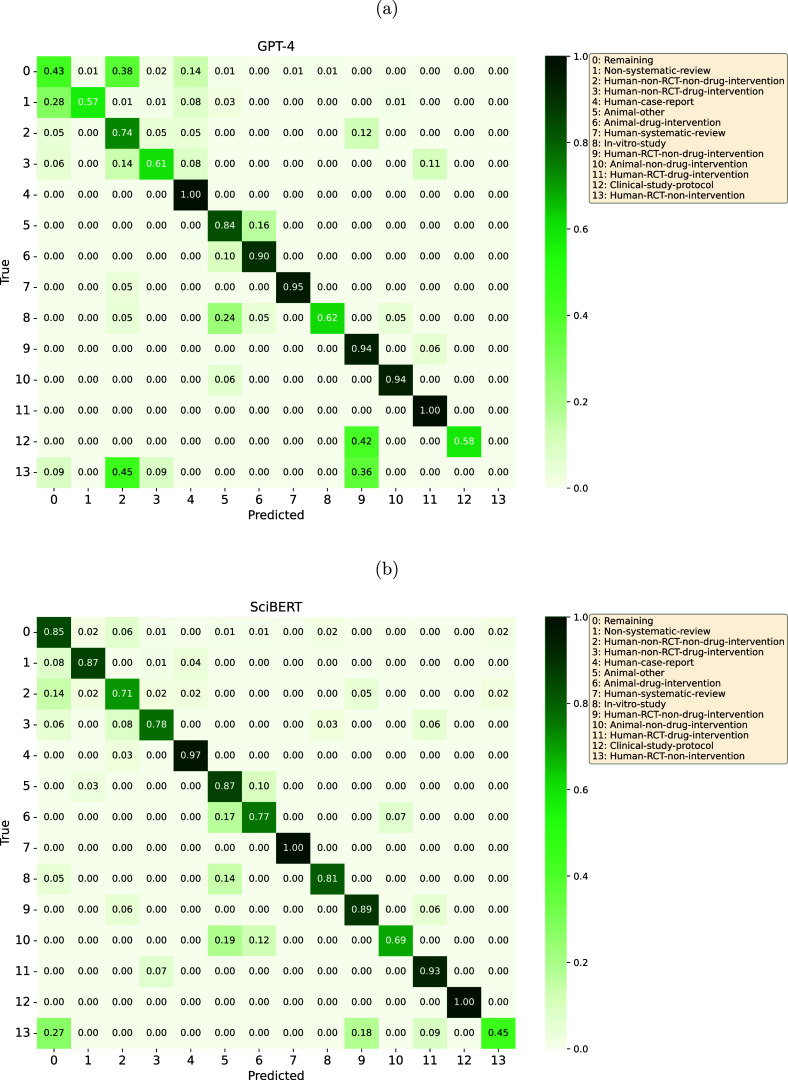
Confusion matrices of the best-performing (a) prompting strategy for the GPT-based model (GPT-4, P2_H_b3, CC) and (b) the BERT-based models (SciBERT).

GPT-4’s most inaccurately predicted classes were *Human-RCT-non-intervention* (0% correctly identified, commonly confused with *Human-non-RCT-non-drug-intervention* and *Human-RCT-non-drug-intervention*), *Non-systematic-review* (57%, commonly confused with *Remaining*), and *Study-protocol* (58%, commonly confused with *Human-RCT-non-drug-intervention*). In addition, the class *Remaining* was commonly confused with *Human-non-RCT-non-drug-intervention* (Figure 3). We did not observe an association between class sizes and model performance.

**Figure 4:**
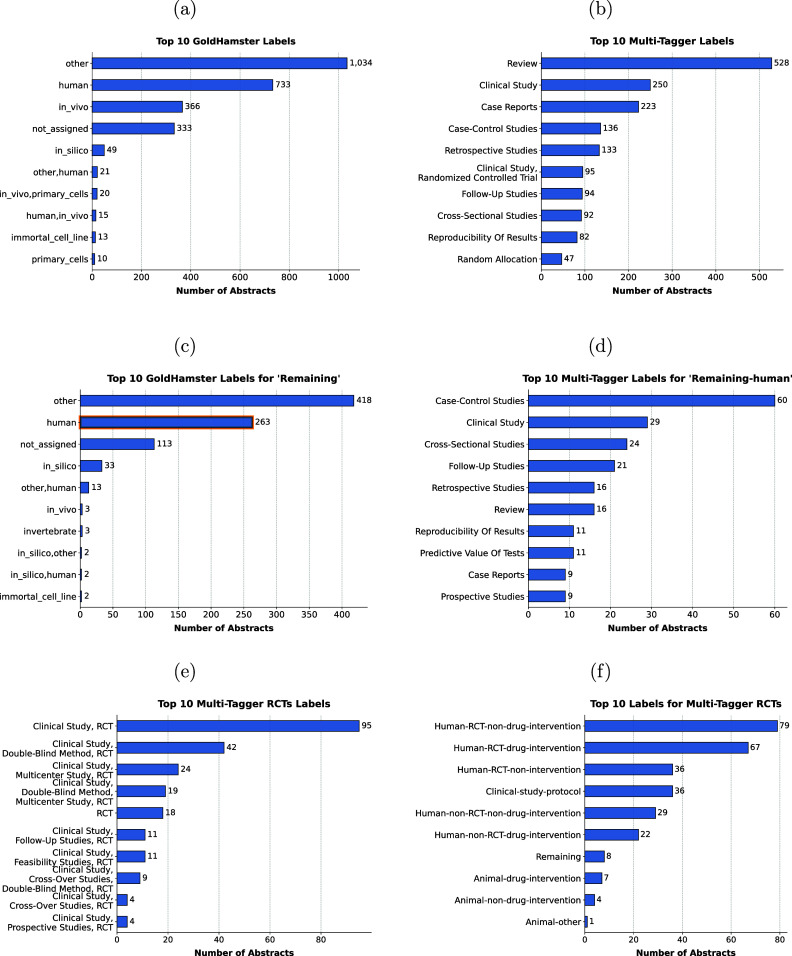
Overview of top ten predicted labels based on the GoldHamster or Multi-Tagger corpus for (a, b) the full dataset, (c) the abstracts annotated as *Remaining* in our corpus, and (d) the abstracts annotated as *Remaining* in our corpus and as *human* by GoldHamster, highlighted in orange in (c). Subfigure (e) shows the top ten labels from Multi-Tagger containing *Randomized Control Trials*, abbreviated as RCT, and (f) the corresponding assigned labels to this set of articles in our dataset.

### 3.3 BERT models

The fine-tuned BERT models also performed almost perfectly in the binary text classification task (i.e., “Animal” versus “Other”) with F1-scores up to 0.99 (Table S6 in the Supplementary Material). And, in contrast to GPT models, this performance remained high for multi-class classification (considering all 14 study classes) with F1-scores between 0.80 and 0.84 for different BERT models (Table 3), with SciBERT being the top-performing model.

SciBERT’s most inaccurately predicted classes were *Human-RCT-non-intervention* (45% correctly identified, commonly confused with *Remaining* and *Human-RCT-non-drug-intervention*) and *Animal-non-drug-intervention* (69%, commonly confused with the classes *Animal-other* or *Animal-drug-intervention*) (Figure 3). The size of the classes (ranging from 54 to 858) was not associated with model performance.

### 3.4 GPT versus BERT

Overall, SciBERT (the best performing BERT model) outperformed both GPT-3.5 and GPT-4 (Table 4 and Figure 2). Despite overlapping confidence intervals, there was a clear trend that SciBERT also outperformed GPT at class-level for most classes, except that GPT-4 outperformed SciBERT for *Human-RCT-drug-intervention*, *Animal-non-drug-intervention,* and *Animal-drug-intervention* (Figure 2 and Figure S2 in the Supplementary Material).

**Table 4:** Top performing models and strategies among the GPT and BERT models, evaluated in the multi-class classification task

Model (Prompt, Concept)	Precision (CI)	Recall (CI)	F1-Score (CI)
GPT-4 (P2_H_b3, CC)	0.741 (0.699, 0.772)	0.644 (0.603, 0.684)	0.645 (0.603, 0.686)
GPT-3.5 (P2_H_b3, CC)	0.617 (0.561, 0.655)	0.551 (0.507, 0.592)	0.540 (0.495, 0.583)
SciBERT	0.843 (0.807, 0.868)	0.839 (0.805, 0.869)	0.839 (0.805, 0.868)

Abbreviations: “P2_H_b3” is hierarchical prompt P2, “P2_HIERARCHY,” whose prediction was based upon the predictions of binary prompt P3.

### 3.5 Comparison to related work

The MeSH-based approach performed well in capturing nearly all animal articles (high recall). However, it mislabeled more articles as *Animal*, which resulted in lower precision. As a result, its overall F1-score was lower than that of the ML-based models (Table S5 in the Supplementary Material).

The *GoldHamster* model most frequently predicted the labels *Other*, *Human*, and *In vivo*. The model returned no label for around 300 abstracts when the model was uncertain about the correct classification (Figure 4a). For the abstracts in our *Remaining* category, the *GoldHamster* model likewise assigned the general category *Other* most often, followed by *Human* and *not assigned* (Figure 4c). Notably, the category *In silico* was also present. In the binary classification task, the model trained on the *GoldHamster* corpus performed comparably to SciBERT trained on our dataset (Table S5 in the Supplementary Material). Although its precision for *Animal* was slightly lower, its recall was higher, likely because multiple labels for some studies led to both more false positives and a broader capture of relevant studies.

When keeping only the *Multi-Tagger* labels whose scores exceed the provided optimal threshold, there were more than 1000 studies without an assigned label (S1a). To mitigate this, we selected the label with the highest score, when the probability for none of the labels achieved the Multi-Tagger defined thresholds. The top predicted labels over the whole datasets were *Review*, *Clinical Study,* and *Case Report* (Figure 4b). *Case-Control Studies*, *Cross-Sectional Studies,* and *Retrospective Studies* were the most common classifications for the articles we labeled as *Remaining* (S1b).

We were also interested in the case where the model trained on the GoldHamster dataset predicted *Human* study type, while our accepted label was *Remaining*. The most frequent labels predicted by *Multi-Tagger* for those 263 studies were *Case-Control Studies*, *Clinical Study,* and *Cross-Sectional Studies* (Figure 4d). Furthermore, articles assigned an RCT label by *Multi-Tagger* were also mostly identified as such in our dataset (Figure 4e and f).

## 4 Discussion

### 4.1 Main findings

This study aimed to use GPT-based LLMs for classification of scientific study types and to compare these models with smaller fine-tuned BERT-based language models. We find that GPT models, including the newer GPT-4, show only mediocre performance in classifying scientific study types, such as RCTs or animal studies. The greatest performance boost, although still moderate, was observed using contextual clues by providing the actual annotation guidelines to GPT. BERT models consistently outperformed GPT models by a substantial margin, reaching F1-scores above 0.8 for most study classes.

### 4.2 Findings in the context of existing evidence

We present a new human-annotated corpus of scientific abstracts tailored to various types of systematic reviews. This corpus includes abstracts relevant for clinical systematic reviews that often involve RCTs, e.g., of therapeutic interventions.^47^ Additionally, it covers study types pertinent to preclinical systematic reviews,^48^ such as animal and in-vitro studies, with sub-classifications specifically for research into therapeutic interventions. This provides much greater granularity than existing corpora,^16^ making it a resource for, e.g., systematic reviews of preclinical studies.^19^

Transformer-based models like BERT, i.e., a type of deep learning model architecture originally developed for text processing, have been used in the screening of scientific articles by automatically classifying study types,^17^ reaching high sensitivity and specificity for differentiating a smaller number of different study types.^16^ BERT-models are pre-trained on a vast corpus of text and subsequently fine-tuned to perform specific tasks like text classification.^35^ Consequently, the BERT family of models has become foundational when fine-tuning models for specific applications like classification, and for a target domain like medicine.^41^ For instance, BioBERT, pre-trained on extensive biomedical corpora, excels in biomedical text mining tasks and can be adapted into even more specialized models.^38^ Our findings show that SciBERT may perform best in terms of the F1-score, though the small differences and overlapping confidence intervals compared to other BERT models preclude firm conclusions. Interestingly, it has been shown that combining different BERT models in an ensemble could enhance the performance of automated text classification even further, achieving sensitivities for text classification as high as 96%.^49^

As expected, GPT-4 showed better performance in study type classification than its predecessor, GPT-3.5, although its overall performance remained moderate. Notably, even advanced prompting strategies^32^^,^ ^50^ that incorporated Contextual Clues (providing annotation guidelines) and Chain-of-Thought (explicit step-by-step reasoning) provided only marginal improvements in performance. For multi-class classification, the more traditional BERT models consistently outperformed the GPT models across all performance metrics in our sample. This is particularly striking given the exceptional abilities of GPT models in natural language understanding and generation across various applications. For instance, GPT models have shown remarkable results in the USMLE—a comprehensive three-step exam that evaluates clinical competency for medical licensure in the United States^28^^,^ ^29^^,^ ^51^—and have even surpassed human performance in text annotation tasks.^52^ This serves as a reminder that, being generative models, their impressive language generation abilities may not necessarily predict good performance on classification tasks, particularly multi-class and domain-specific tasks. Notably, GPT-models have been used with some success to automatically perform title and abstract screening for systematic reviews,^53^^–^ ^56^ though it has not yet reached the level of fully replacing even one human reviewer, let alone two.^53^ We also considered fine-tuning open-weight LLMs. However, GPT models already offer a strong baseline and fine-tuning is costly and time-consuming. With limited academic resources, we chose GPT for its competitive results out of the box.

We showed that using MeSH terms to differentiate between animal and other study types, resulted in more false positive animal-study classifications. This aligns with findings that some MeSH terms are not good discriminators for study types, due to their broader assignment.^13^ We did not experiment with integrating MeSH terms into the text for fine-tuning/GPT prediction, as similar approaches were extensively evaluated in related work and showed no performance improvement.^13^

A comparison with the labels predicted by the *GoldHamster* model showed high agreement in identifying animal studies. At the same time, the fine-grained annotations in our dataset and the *GoldHamster* corpus appear complementary. Notably, our work differentiates animal studies by intervention type (drug versus non-drug), whereas the *GoldHamster* corpus also covers non-animal study models, such as in-silico approaches. The *GoldHamster* model assigned a large proportion of studies to the non-specific *Other* class, suggesting additional relevant categories that are not captured by either dataset. Using the *Multi-Tagger* annotations revealed that these unlabeled studies mostly concern clinical studies in humans, including case-control or observational studies, which was not the focus in our dataset. For articles annotated as RCTs, *Multi-Tagger* can provide more detail for the study design, while our annotations help differentiate between RCTs of different intervention types. Applying the optimal *Multi-Tagger* thresholds would leave over 1,000 studies in our corpus unlabeled, given *Multi-Tagger*’s exclusive focus on human-relevant articles. Those observations suggest a promising future research direction to merge those related datasets to create a more comprehensive resource.

### 4.3 Limitations

Our study has a number of limitations: First, the natural distribution of study types in PubMed resulted in an imbalanced dataset, with, e.g., study protocols or non-interventional RCTs being underrepresented. The *Remaining* class, which includes all studies not fitting into other specified categories, was almost three times larger than the second largest class. Although we maintained this distribution to reflect real-world scenarios, we manually enriched each of the smaller classes with an additional 50 samples to partially mitigate the uneven class distribution and resolve the issue of severely underrepresented classes. Future research could assess how a more balanced class distribution could affect BERT model performance.

Second, although we employed various advanced prompting strategies, we did not explore role modeling with GPT that might enhance its effectiveness (e.g., “You are a systematic reviewer”). Future studies could also look into lightweight model adaptation methods like delta-tuning methods,^57^ such as BitFit^58^ and adapters^59^ to optimize performance.

Third, our use of GPT models was limited to GPT-3.5-turbo and GPT-4-turbo-preview, excluding other available API versions. Fourth, it was decided that model calibration (i.e., aligning the model’s confidence scores with actual accuracy) would exceed the technical scope of the study. We recognize, however, that the calibration between confidence and performance^60^ would be essential for studies focusing on the creation of robust and optimized models.

Finally, limitations in the study scope and generalizability should be mentioned. While not a limitation of this study specifically, the applicability of automated study-type classification may be restricted to biomedical research. Fields, such as education or ecology, may lack clearly defined study types, limiting the usefulness of such approaches. Furthermore, our sampled articles were retrieved using a query focused on the neuroscience domain. While our aim was to develop study type definitions that are broadly useful for biomedical text mining, the resulting corpus is specific to neuroscience. However, we believe that the key features a classification model uses to identify study types are largely independent of the disease domain. Nonetheless, further experiments are required to assess the generalizability of our approach across other areas of biomedical research.

### 4.4 Strengths

Our study has two main strengths: First, we introduce a highly granular, dual-annotated abstract corpus designed for fine-tuning LLMs. This allows for more precise adaptations to specific tasks within text classification. Second, we conducted a comprehensive comparison of state-of-the-art GPT models, incorporating advanced prompting strategies, against a range of established BERT models. This approach enhances capabilities beyond those of PubMed indexing or MeSH terms.

### 4.5 Conclusions

Our study demonstrates that LLMs, particularly those based on BERT, can be used for classification of study types for systematic reviews. These models are especially effective for this task. Specifically, trimming a reference library prior to formal abstract screening with such an approach could dramatically reduce human workload during the abstract screening phase of systematic reviews. As the volume of scientific publications continues to grow, employing such tools will be critical in keeping abreast with scientific evidence.

## Supporting information

Emilova Doneva et al. supplementary material Emilova Doneva et al. supplementary material

## Data Availability

All data and code that support the findings of this study are available in our public GitHub repository (https://github.com/Ineichen-Group/StudyTypeTeller). For any questions regarding the data, metadata, or code analysis, contact the corresponding author, S.E.D.

## Supplementary material

The supplementary material for this article can be found at https://doi.org/10.1017/rsm.2025.10031.
