# Supplementary material for: StudyTypeTeller—Large language models to automatically classify research study types for systematic reviews
Source: Res Synth Methods. 2025 Sep 11;16(6):1005–24. doi: 10.1017/rsm.2025.10031 (PMC12657658; doi:10.1017/rsm.2025.10031)

7. Supplementary data

a Annotation guidelines study type labels

a.1 Study classes

Studies are assigned one single class out of 15 predefined classes, shown in Table S1.

a.2 Inclusion and exclusion criteria

We included studies in the field of neuroscience/neurology/psychiatry.

We excluded studies without abstract and studies where non-roman letters made up a substantial part of the text (i.e., except for the use of certain non-roman letters and characters in scientific formula).

a.3 Definition of 'intervention', 'drug-intervention' and 'non-drug-intervention'

The working definition for 'intervention' is the following: An 'intervention' includes a treatment, procedure, or other action taken to prevent or treat disease, or improve health in other ways.

We separate 'Non-drug-interventions' from 'drug-interventions' according to the following definition: For the annotation on drug-based, respective chemical-based therapies, we follow the guidelines of the CHEMDNER corpus for annotating chemical mentions [61], as well as [62]. The basic rule for chemical entity annotation is that the chemical should have a specific structure. Non-drug-interventions include any therapeutic intervention not classifiable as drug, e.g., radiation therapy, physical therapy or complex diets. This also includes other substances, that cannot be associated to a clear molecular structure, such as olive oil, herbal extracts, cannabis, tea and nanoparticles (and others).

a.4 Guidelines for annotation of clinical trials

Studies which re-analyze data from former clinical trials will be labeled as the respective clinical trial.

Pharmakokinetic studies will be labeled as the respective drug-intervention study.

Studies on long-term adverse events of therapies will be labeled as the respective clinical trial. This applies only if the therapy is mentioned specifically. E.g., a non-randomized study testing aspirin for dementia would be classified as *non-RCT-drug-intervention* whereas a study on carotid artery sclerosis after irradiation would be classified as *Remaining* since irradiation is an non-specific description of a therapy.

a.5 General guidelines for annotation

The labels are presented in a hierarchical order as presented in Table S1 (column 'hierarchy'). Specifically, when multiple labels were applicable to an abstract, only the label

with the highest priority according to the hierarchy would be assigned.

b Shortened Annotation guidelines by study class

b.1 Definition of Interventions

'Intervention' includes treatments, procedures, or actions to prevent or treat disease. *Non-drug-interventions* encompass therapies without a defined molecular structure.

b.2 Guidelines for Clinical Trials

Studies re-analyzing clinical trial data labeled as respective clinical trials. Pharmacokinetic studies labeled as drug-intervention studies. Long-term adverse events studies labeled as clinical trials if the therapy is mentioned specifically.

b.3 General Annotation Guidelines

Labels are hierarchical; select the highest applicable label. Refer to the accompanying table (Table S2) for the hierarchy of labels. 'Remaining' is used for studies outside neuroscience/psychiatry, clinical trials with unspecific therapies, and uncertainties.

b.4 Study Labels

Labels defined for different study types; choose the most specific applicable label.

This part of the shortened annotation guidelines was shortened with the help of ChatGPT [63] before manual editing.

c Mathematical definitions of performance evaluation metrics

We define the performance metrics used in our evaluation as follows:

- **Accuracy**:

$$\text{Accuracy} = \frac{\text{TP} + \text{TN}}{\text{TP} + \text{TN} + \text{FP} + \text{FN}} \tag{1}$$

  where TP, TN, FP, and FN represent the true positives, true negatives, false positives, and false negatives, respectively.

- **Precision**:

$$\text{Precision} = \frac{\text{TP}}{\text{TP} + \text{FP}} \tag{2}$$

- **Recall (Sensitivity)**:

$$\text{Recall} = \frac{\text{TP}}{\text{TP} + \text{FN}} \tag{3}$$

- **F1-Score (Binary Case)**:

$$\text{F1} = 2 \cdot \frac{\text{Precision} \cdot \text{Recall}}{\text{Precision} + \text{Recall}} \tag{4}$$

- **Weighted Precision, Recall, and F1-Score**:

$$\text{Weighted Metric} = \sum_{i=1}^{C} w_i \cdot \text{Metric}_i \tag{5}$$

  where

$$w_i = \frac{n_i}{\sum_{j=1}^{C} n_j} \tag{6}$$

  represents the weight for class i, proportional to the number of instances n_i in that class, and Metric_i is the metric (Precision, Recall, or F1) computed for class i. C is the total number of classes.

- **Confidence Intervals (based on library [46])**:
  - For **binary precision and recall**, confidence intervals are derived using the binomial proportion method as implemented in `proportion_confint`.
  - For **binary F1-scores**, variance is estimated using the delta method as described by Takahashi et al. [45].
  - For **weighted metrics**, confidence intervals are computed using bootstrapping, where the test set is resampled with replacement and the metric is recalculated multiple times, as implemented in `scipy.stats.bootstrap`. We used the bias-corrected and accelerated (BCa) bootstrap method with 9999 resamples and a confidence level of 95%.

d BERT-Based Models

- **bert-base-uncased (BERT-base)** [35]: The first in the series of BERT models, it has been pretrained on unlabeled text in the English language from Wikipedia and BooksCorpus (introduced in the following paper [64]), using a masked language modeling (MLM) objective. First released in 2018, it employs joint conditioning on both left and right context in all layers.
- **allenai/scibert_scivocab_uncased (SciBERT)** [37]: SciBERT follows the same architecture as BERT but is instead pretrained on the full-texts and abstracts of computer science and biomedical papers from the corpus of Semantic Scholar.
- **dmis-lab/biobert-v1.1 (BioBERT)** [38]: A domain-specific BERT model pre-trained on large-scale biomedical corpora. BioBERT has been pre-trained on biomedical domain corpora of PubMed abstracts and PMC full-text articles.
- **emilyalsentzer/Bio_ClinicalBERT (ClinicalBERT)** [39]: Bio_ClinicalBERT has been initialized from BioBERT and additionally trained on all clinical notes from MIMIC-III ($\sim$ 880M words)[65].
- **microsoft/BiomedNLP-BiomedBERT-base-uncased-abstract (BiomedBERT)** [40]: This variety of BiomedBERT is presented as a tool specifically for the domain of biomedical research. It has been pretrained from scratch using abstracts from PubMed.
- **microsoft/BiomedNLP-PubMedBERT-base-uncased-abstract-fulltext (PubMed-BERT)** [41]: Building upon BiomedBERT, PubMedBERT has been pretrained from scratch using abstracts from PubMed as well as full-text articles from PubMed-Central.
- **michiyasunaga/BioLinkBERT-base (BioLinkBERT)** [42]: BioLinkBERT is a biomedical domain-specific language model pretrained on PubMed with citation links. It leverages LinkBERT, a pretraining method that leverages hyperlinks between documents.

e Hyperparameter optimization

Hyperparameter tuning was carried out using Weights & Biases platform [66]. As outlined in Section 5.3, it was performed on a subset of the initial dataset.

f Missing Author Keywords

Figure 5 illustrates the percentage of studies missing author keywords across different label categories.

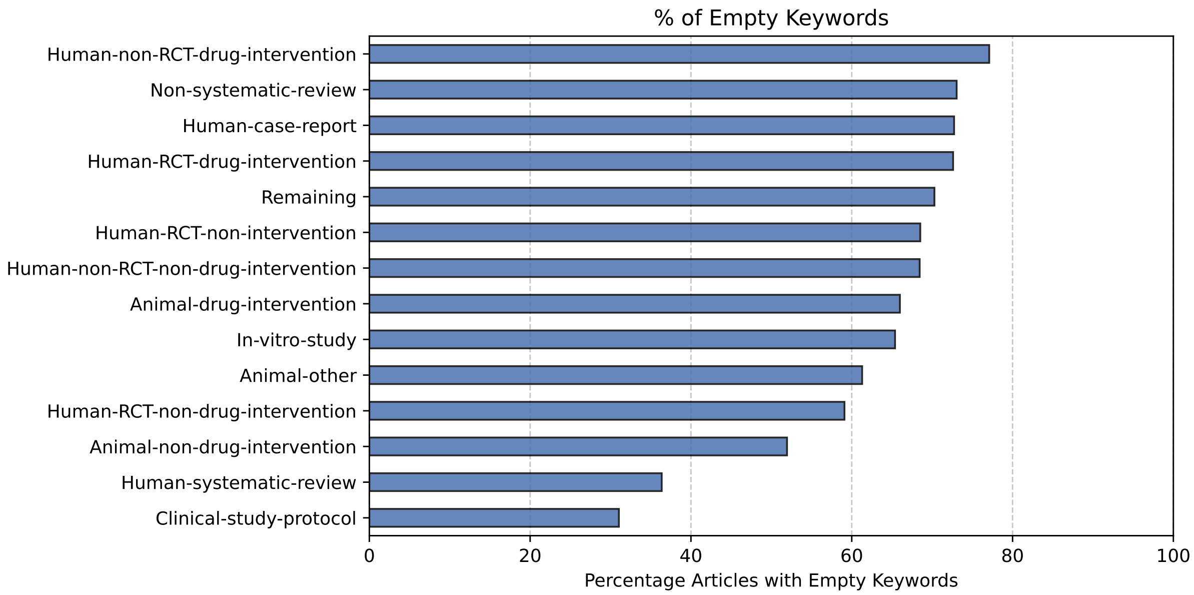

Figure 5: Overview of percentage missing keywords for the studies in each label category.

g Dataset Enrichment Effect on BERT Performance

Dataset enrichment led to a substantial improvement of the BERT-based models' performance on the multiclass classification task. See the below confusion matrix for SciBERT's performance on the non-enriched dataset.

(a) SciBERT's performance on the non-enriched dataset. A comparison with the same model's performance on the enriched dataset (Fig. 3) shows the latter's increased performance across most classes, particularly prominent across the minority classes of Clinical-study-protocol and Human-RCT-non-intervention.

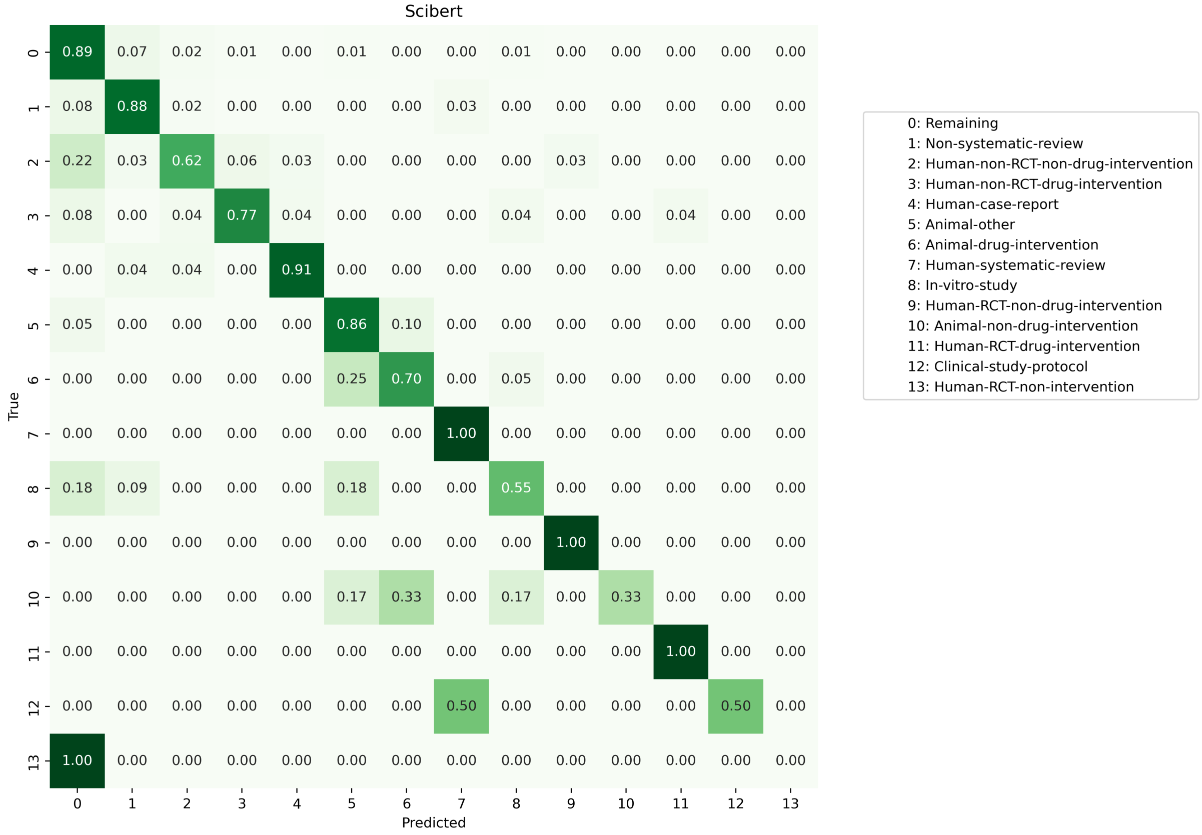

h Prompts for the binary classification task ('Animal' versus 'Other')

```
{
  "prompts": [
    {
      "id": "P1",
      "text": "Classify this text, choosing one of these labels: 'ANIMAL' if the text is
          related to animal, and 'OTHER' for any other study type. Respond in json format
           with the key: gpt_label.",
      "strategy_type": "zero_shot"
    },
    {
      "id": "P2",
      "text": "Please classify the provided text by selecting the appropriate label. Use
          the label 'ANIMAL' if the text discusses anything related to animals, and '
          OTHER' for all other types of content. Provide your response in JSON format
          using the key gpt_label. For instance, if the text is about animals, your
          response should be {gpt_label: ANIMAL}.",
      "strategy_type": "zero_shot"
    },
    {
      "id": "P3",
      "text": "Please classify the provided text by selecting the appropriate label. Use
          the label 'ANIMAL' if the text discusses anything related to animal research,
          such as summary of animal studies, studies testing interventions on animals,
          and any other studies involving animals. Use 'OTHER' for all other research
          types, such as human clinical trials, reviews, or theoretical studies. Provide
          your response in JSON format using the key gpt_label. For instance, if the text
           is about animals, your response should be {gpt_label: ANIMAL}.",
      "strategy_type": "zero_shot"
    }
  ]
}
```

i Prompts for the hierarchical study classification

```
{
  "prompts": [
    {
       "id": "P1_HIERARCHY",
      "text_animal": "Classify this text, choosing one of these labels: Animal-systematic
          -review, Animal-drug-intervention, Animal-non-drug-intervention, Animal-other.
          Respond in json format with the key: gpt_label.",
      "text_other": "Classify this text, choosing one of these labels: Clinical-study-
          protocol, Human-systematic-review, Non-systematic-review, Human-RCT-non-drug-
          intervention, Human-RCT-drug-intervention, Human-RCT-non-intervention, Human-
          case-report, Human-non-RCT-non-drug-intervention, Human-non-RCT-drug-
          intervention, In-vitro-study, Remaining. Respond in json format with the key:
          gpt_label.",
      "strategy_type": "zero_shot"
    }
  ]
}
```

The second hierarchical prompt, referred to in this paper as 'P2_HIERARCHY', is available in our public GitHub repository (https://github.com/Ineichen-Group/StudyTypeTeller/blob/main/models/gpt/prompts/prompt_strategies_hierarchical.json)

j Prompts for the multi-class classification task

All multi-class prompts are available in our public GitHub repository (https://github.com/Ineichen-Group/StudyTypeTeller/blob/main/models/gpt/prompts/prompt

_strategies.json). Displayed here are two examples of multi-class prompts, P1 and P4_1.

```
{
  "prompts": [
    {
      "id": "P1",
      "text": "Classify this text, choosing one of these labels: Clinical-study-protocol,
          Human-systematic-review, Non-systematic-review, Human-RCT-non-drug-
         intervention, Human-RCT-drug-intervention, Human-RCT-non-intervention, Human-
         case-report, Human-non-RCT-non-drug-intervention, Human-non-RCT-drug-
         intervention, Animal-systematic-review, Animal-drug-intervention, Animal-non-
         drug-intervention, Animal-other, In-vitro-study, Remaining. Respond in json
         format with the key: gpt_label.",
      "strategy_type": "zero_shot"
    },
    {
      "id": "P4_1",
      "text": "Classify the following text by choosing the most appropriate of theses
         labels while adhering to the list of annotation guidelines below: Clinical-
         study-protocol, Human-systematic-review, Non-systematic-review, Human-RCT-non-
         drug-intervention, Human-RCT-drug-intervention, Human-RCT-non-intervention,
         Human-case-report, Human-non-RCT-non-drug-intervention, Human-non-RCT-drug-
         intervention, Animal-systematic-review, Animal-drug-intervention, Animal-non-
         drug-intervention, Animal-other, In-vitro-study, Remaining. Respond in JSON
         format with the key: gpt_label.\n\nInformation for Classification:\n1.
         Definition of Interventions: 'Intervention' includes treatments, procedures, or
          actions to prevent or treat disease.'Non-drug-interventions' encompass
         therapies without a specific molecular structure.\n2. Guidelines for Clinical
         Trials: Studies re-analyzing clinical trial data labeled as respective clinical
          trials. Pharmakokinetic studies labeled as drug-intervention studies. Long-
         term adverse events studies labeled as clinical trials if the therapy is
         mentioned specifically.\n3. General Annotation Guidelines: Labels are
         hierarchical; select the highest applicable label. Refer to the numbers
         accompanying  the labels for the hierarchy. 'Remaining' is used for studies
         outside neuroscience/psychiatry, clinical trials with unspecific therapies, and
          uncertainties. \n4. Study Labels: Labels defined for different study types;
         choose the most specific applicable label.\n\nLabels and Guidelines:\n1. Study
         protocol: A study protocol for a clinical trial or systematic review.\n2. Human
         , systematic review: A structured research summary including human data.\n3.
         Non-systematic review: A text reviewing a certain topic in a non-systematic way
         .\n4. Human, RCT non-drug-intervention: A randomized controlled trial of a non-
         drug therapeutic intervention in human participants.\n5. Human, RCT drug-
         intervention: A randomized controlled trial of a drug intervention in human
         participants.\n6. Human, RCT non-intervention: A randomized controlled trial
         not testing any therapeutic intervention.\n7. Human, case report: A clinical
         study reporting findings of one single human individual.\n8. Human, non-RCT non
         -drug-intervention: Any non-RCT clinical study of a non-drug therapeutic
         intervention in human participants.\n9. Human, non-RCT drug-intervention: Any
         non-RCT clinical study of a drug intervention in human participants.\n10.
         Animal, systematic review: A structured research summary including animal data
         .\n11. Animal, non-drug-intervention: An animal study testing a non-drug
         therapeutic intervention.\n12. Animal, drug-intervention: An animal study
         testing a drug.\n13. Animal, other: Any animal study not testing a therapeutic
         intervention.\n14. In-vitro study: An experimental study conducted outside a
         living organism.\n15. Remaining: Any remaining study type not covered by the
         above definitions.\n\nYour task is to read the journal name, title and abstract
          provided, apply these guidelines, and determine the most accurate
         classification for the study. Ensure your response adheres to the specified
         format for submission.",
      "strategy_type": "with_shortened_annotation_guidelines"
    }
  ]
}
```

Table S1: Annotation guidelines for study classes.

first word	label	working definition used (applies to title and/or abstract of study)	hierarchy
	study protocol	A publication outlining the design, objectives, methodology, statistical considerations or ethical considerations of a clinical research study or a systematic review/meta-analysis [67]. Explicit mention of 'protocol'.	1
human	systematic review	A structured and reproducible research summary of human participants. This also includes systematic reviews covering both human subjects and animals. An explicit mention of 'systematic review', 'we systematically review', 'meta-analysis' and/or 'we meta-analyze' in the title or abstract. Alternatively, the mention of at least 2 biomedical literature databases such as 'PubMed', 'Embase', 'Cochrane Library', 'Web of Science', 'Scopus' (or others) in the abstract and at least 2 authors would qualify as a systematic review.	2
	non-systematic review	A text reviewing/discussing a certain topic, can be very broad and includes editorials, perspectives and other types of texts with the goal to discuss any matter related to neuroscience/psychiatry. Does not generate original data but discusses previously published data. An explicit mention of 'review', 'expert review', 'traditional review', 'literature review', 'review of literature', 'expert consensus' or 'we summarize'.	3
human	RCT non-drug-intervention	A scientific experiment in the form of a clinical trial with the distinguishing feature that participants are randomized into experimental groups. This study type needs a specific mention of randomization of a human population. Concretely, the aim of the study must be to test a non-drug treatment (e.g., radiation therapy, surgery, physical therapy, nanoparticles) in randomized fashion.	4
	RCT drug-intervention	A scientific experiment in the form of a clinical trial with the distinguishing feature that participants are randomized into experimental groups. This study type needs a specific mention of randomization of a human population. Concretely, the aim of the study must be to test a drug (e.g., aspirin, risperdal, lorazepam) in randomized fashion.	5
	RCT non-intervention	A scientific experiment in the form of a clinical trial with the distinguishing feature that participants are randomized into experimental groups. This study type needs a specific mention of randomization of a human population. Any RCT not annotated as 'RCT-drug-intervention' or 'RCT-non-drug-intervention' should be classified under this category.	6
	case report	A study type reporting clinical/imaging findings of one single human individual, e.g., by explicit mentioning of 'case' or 'case report'. Case series with more than 1 participants shall not be classified under this category.	7
	non-RCT non-drug-intervention	A clinical study without explicit mention of randomization testing a non-drug intervention, e.g., observational or cohort studies. Also includes studies assessing participants which were sampled from a previous RCT.	8
	non-RCT drug-intervention	A clinical study without explicit mention of randomization testing a drug intervention, e.g., observational or cohort studies. Also includes studies assessing participants which were sampled from a previous RCT.	9
animal	systematic review	A structured and reproducible research summary of animal studies only. An explicit mention of 'systematic review', 'we systematically review', 'meta-analysis' and/or 'we meta-analyze' in the title or abstract. Alternatively, the mention of at least 2 biomedical literature databases such as 'PubMed', 'Embase','Cochrane Library', 'Web of Science', 'Scopus' (or others) and at least 2 authors would qualify as a systematic review.	10
	non-drug-intervention	Any study type testing a non-drug intervention such as radiation therapy, surgery, physical therapy or nanoparticles in animals, including experimental studies but also studies in companion animals (pets).	11
	drug-intervention	Any study type testing a drug intervention in animals, including experimental studies but also studies in companion animals (pets).	12
	other	Any animal study not testing a therapeutic intervention, e.g., pathomechanistic studies, methods development, case series/reports (veterinary studies), studies testing diagnostic procedures in animals. This also includes exposure studies in animals (e.g., farm animals being exposed to certain environmental toxins).	13
	in-vitro-study	Any experimental research conducted outside a living organism, i.e., on isolated cells, tissues, organs, enteroids or organoids under controlled conditions.[68] This excludes genetic studies and pathological studies in tissue from a clearly defined patient/control cohort.	14
	remaining	Any other study which does not fit in any of the categories defined above. E.g., studies about medical history, epidemiology or other. Also includes studies which raters were uncertain how to label, clinical trials with unspecific mention of therapies (e.g., 'antiepileptica', studies published in the 'Journal of Visualized Experiments' (JoVE) and studies which are outside the realm of neuroscience/psychiatry.	15

Please note that we have 15 labels in these annotation guidelines, but only 14 throughout the paper because of missing examples of *Animal-systematic-review*, see Section 3.2.3.

Table S2: Shortened annotation guidelines for study classes.

**first word**	**label**	**working definition used**	**hier-archy**
	study protocol	A study protocol for a clinical trial or systematic review.	1
human	systematic review	A structured research summary including human data.	2
	non-systematic review	A text reviewing a certain topic in a non-systematic way.	3
human	RCT non-drug-intervention	A randomized controlled trial of a non-drug therapeutic intervention in human participants.	4
	RCT drug-intervention	A randomized controlled trial of a drug intervention in human participants.	5
	RCT non-intervention	A randomized controlled trial not testing any therapeutic intervention.	6
	case report	A clinical study reporting findings of one single human individual.	7
	non-RCT non-drug-intervention	Any non-RCT clinical study of a non-drug therapeutic intervention in human participants.	8
	non-RCT drug-intervention	Any non-RCT clinical study of a drug intervention in human participants.	9
animal	systematic review	A structured research summary including animal data.	10
	non-drug-intervention	An animal study testing a non-drug therapeutic intervention.	11
	drug-intervention	An animal study testing a drug.	12
	other	Any animal study not testing a therapeutic intervention.	13
	in-vitro-study	An experimental study conducted outside a living organism.	14
	remaining	Any remaining study type not covered by the above definitions.	15

Please note that we have 15 labels in these annotation guidelines, but only 14 throughout the paper because of missing examples of *Animal-systematic-review*, see Section 3.2.3.

Table S3: Text Statistics: abstract statistics across all splits, with the values representing the number of word-level tokens.

	Min	Mean	Max
Whole dataset	17	237	1040
**Classes (binary)**			
Other	17	237	1040
Animal	29	244	509
**Classes (multiclass)**			
Animal-drug-intervention	68	237	509
Animal-non-drug-intervention	29	258	459
Animal-other	69	244	468
Clinical-study-protocol	154	318	605
Human-RCT-drug-intervention	58	312	1040
Human-RCT-non-drug-intervention	95	249	574
Human-RCT-non-intervention	124	313	570
Human-case-report	17	162	432
Human-non-RCT-drug-intervention	52	258	605
Human-non-RCT-non-drug-intervention	55	248	682
Human-systematic-review	102	327	928
In-vitro-study	53	237	433
Non-systematic-review	30	185	751
Remaining	24	233	700

Table S4: Hyperparameters used in the fine-tuning of BERT-based models.

**Hyperparameter**	**Value**
Epochs	10
Patience	4
Batch size	8
Max sequence length	256
Loss function	Cross-entropy loss
Warmup steps	10% of total steps
Learning rate	5e-5
Weight decay	5e-4
Optimizer	AdamW

Figure S1: Top 10 predicted labels based on Multi-Tagger (a) if the optimal reported probability threshold is used. In this case the labels are assigned whose scores exceeded the provided optimal threshold. (b) for all studies classified as *Remaining* in our corpus and using the maximum probability label if the threshold none of the labels is met.

(a)

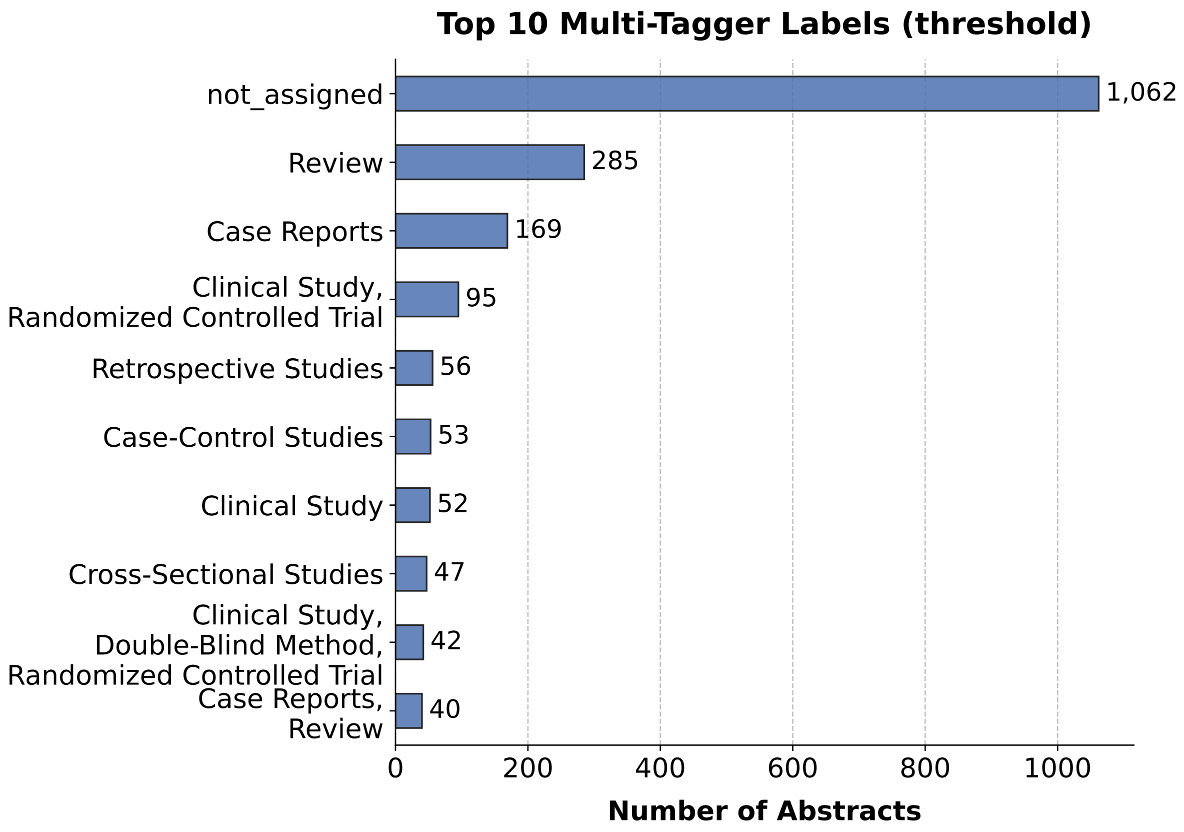

(b)

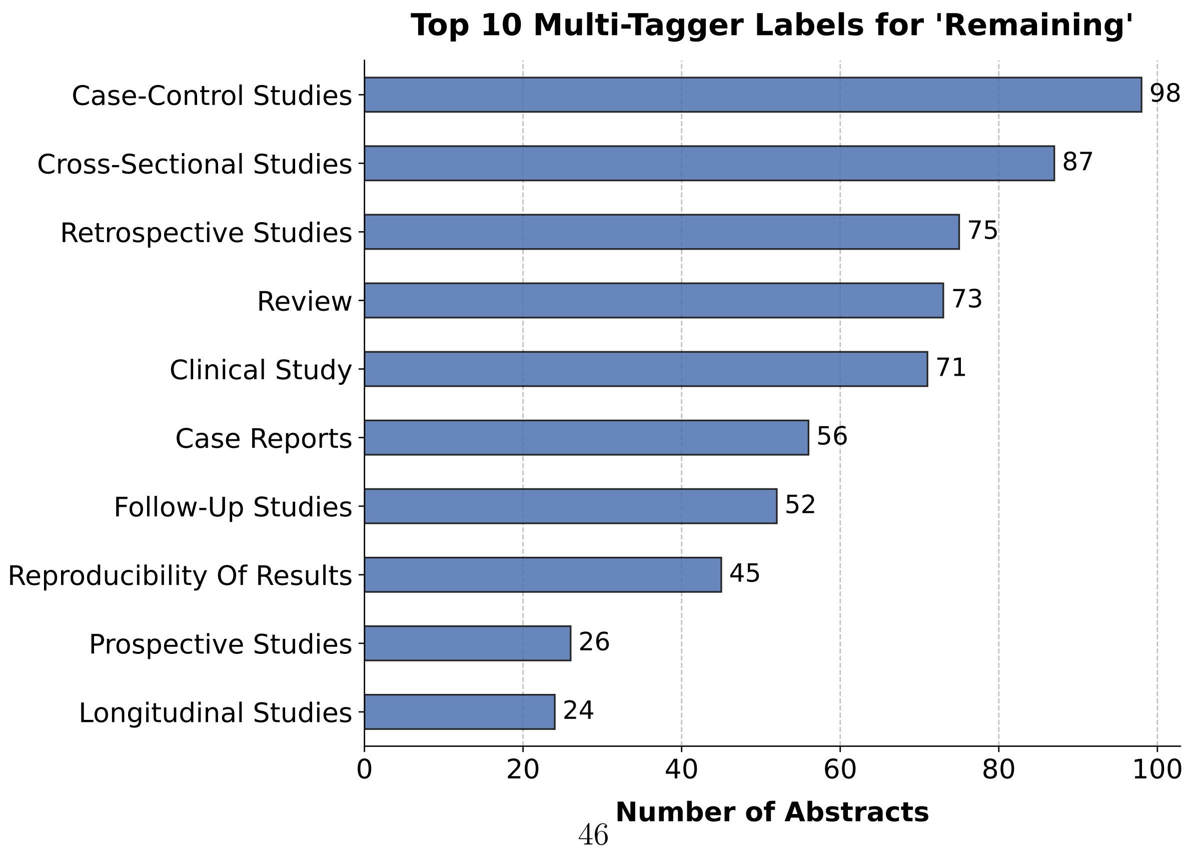

Table S5: Performance comparison of GoldHamster, SciBERT, and MeSH-based predictions for the binary classification task. GoldHamster is a BioLinkBERT model trained on the GoldHamster corpus. MeSH-based predictions assigned the *Animal* label if “animal” appeared in the article’s MeSH terms; otherwise, it was labeled *Other*.

**Model**	**Class**	**Precision (CI)**	**Recall (CI)**	**F1-Score (CI)**
GoldHamster	Other	0.998 (0.987, 1.0)	0.980 (0.963, 0.99)	0.989 (0.978, 1.0)
	Animal	0.894 (0.811, 0.943)	0.987 (0.93, 0.998)	0.938 (0.896, 0.98)
	Weighted Avg	0.983 (0.971, 0.991)	0.981 (0.966, 0.991)	0.982 (0.967, 0.991)
SciBERT	Other	0.989 (0.975, 0.995)	0.996 (0.984, 0.999)	0.992 (0.983, 1.002)
	Animal	0.973 (0.907, 0.993)	0.935 (0.857, 0.972)	0.954 (0.916, 0.992)
	Weighted Avg	0.987 (0.972, 0.993)	0.987 (0.974, 0.994)	0.987 (0.973, 0.994)
MeSH-based	Other	0.999 (0.997, 1.0)	0.935 (0.924, 0.944)	0.966 (0.957, 0.974)
	Animal	0.718 (0.678, 0.755)	0.995 (0.981, 0.999)	0.834 (0.807, 0.862)
	Weighted Avg	0.959 (0.954, 0.963)	0.943 (0.934, 0.952)	0.947 (0.939, 0.955)

Table S6: Performance metrics of binary classification, showing the best-performing BERT models and the prompting strategies of the GPT models. Text in bold indicates the best performance.

Model	Prompt	Precision (CI)	Recall (CI)	F1-Score (CI)
GPT-3.5	P1	0.918 (0.888, 0.94)	0.921 (0.897, 0.942)	0.914 (0.884, 0.938)
GPT-3.5	P2	0.912 (0.883, 0.935)	0.916 (0.890, 0.936)	0.906 (0.875, 0.932)
GPT-3.5	P3	0.948 (0.927, 0.964)	0.946 (0.923, 0.963)	0.947 (0.925, 0.963)
**GPT-4**	**P3**	**0.979 (0.968, 0.988)**	**0.976 (0.961, 0.987)**	**0.976 (0.962, 0.987)**
**SciBERT**		**0.987 (0.972, 0.993)**	**0.987 (0.974, 0.994)**	**0.987 (0.973, 0.994)**
BioLinkBERT		0.985 (0.970, 0.993)	0.985 (0.972, 0.993)	0.985 (0.972, 0.993)
BiomedBERT		0.981 (0.969, 0.990)	0.979 (0.964, 0.989)	0.980 (0.966, 0.989)
GoldHamster*		0.983 (0.971, 0.991)	0.981 (0.966, 0.991)	0.982 (0.967, 0.991)
MeSH-based**		0.959 (0.954, 0.963)	0.943 (0.934, 0.952)	0.947 (0.939, 0.955)

Performance is measured on the enriched dataset inlcuding keywords.
The concept of each binary GPT prompt was zero-shot.
Abbreviations: CI, 95%-confidence interval.
*GoldHamster is a BioLinkBERT model trained on the GoldHamster corpus.
**MeSH-based predictions assigned the *Animal* label if "animal" appeared in the article's MeSH terms; otherwise, it was labeled *Other*.

Table S7: Performance metrics of hierarchical labeling for GPT-3.5-turbo. Text in bold indicates the best performance.

Hierarchical Prompt	Concept	Binary prompt	Precision (CI)	Recall (CI)	F1-Score (CI)
P1_HIERARCHY	zero-shot	P1	0.448 (0.358, 0.529)	0.311 (0.273, 0.352)	0.258 (0.218, 0.301)
P1_HIERARCHY	zero-shot	P2	0.452 (0.36, 0.534)	0.313 (0.273, 0.352)	0.265 (0.224, 0.308)
P1_HIERARCHY	zero-shot	P3	0.515 (0.418, 0.586)	0.346 (0.307, 0.388)	0.295 (0.253, 0.338)
P2_HIERARCHY	CC	P1	0.58 (0.524, 0.621)	0.507 (0.466, 0.551)	0.502 (0.459, 0.546)
P2_HIERARCHY	CC	P2	0.587 (0.529, 0.626)	0.504 (0.463, 0.545)	0.499 (0.456, 0.544)
**P2_HIERARCHY**	**CC**	**P3**	**0.617 (0.561, 0.655)**	**0.551 (0.507, 0.592)**	**0.54 (0.496, 0.585)**
**P2_HIERARCHY**	**GPT-4, CC**	**P3**	**0.741 (0.699, 0.772)**	**0.644 (0.603, 0.684)**	**0.645 (0.603, 0.686)**

Performance is measured on the enriched dataset inlcuding keywords.
'Binary prompt' indicates on what outputs the hierarchical labeling was based, see Section 3.3.2.
Abbreviations: CI, 95%-confidence interval.

Table S8: All performance metrics per prompting strategy for GPT-3.5-turbo on enriched dataset with keywords. Text in bold indicates the best performance.

Prompt	Concept	Precision (CI)	Recall (CI)	F1-Score (CI)
P1	zero-shot	0.454 (0.351, 0.539)	0.331 (0.292, 0.371)	0.261 (0.22, 0.305)
P2	zero-shot	0.45 (0.292, 0.652)	0.279 (0.243, 0.318)	0.203 (0.167, 0.242)
P3_1	CC	0.584 (0.513, 0.64)	0.453 (0.412, 0.494)	0.43 (0.386, 0.476)
P3_2	CC	0.556 (0.487, 0.614)	0.451 (0.41, 0.494)	0.427 (0.382, 0.472)
P4_1	CC	0.563 (0.498, 0.611)	0.434 (0.393, 0.476)	0.416 (0.372, 0.461)
P4_2	CC	0.506 (0.441, 0.556)	0.414 (0.371, 0.457)	0.392 (0.347, 0.437)
P5	CC	0.608 (0.54, 0.65)	0.511 (0.466, 0.552)	0.498 (0.452, 0.542)
**P6**	**CC**	**0.591 (0.536, 0.631)**	**0.541 (0.5, 0.584)**	**0.532 (0.487, 0.575)**
P7	CoT	0.467 (0.334, 0.589)	0.305 (0.266, 0.345)	0.229 (0.19, 0.269)
P9	CoT + CC	0.542 (0.453, 0.616)	0.41 (0.371, 0.453)	0.375 (0.332, 0.42)
P9_1	CoT + CC	0.521 (0.444, 0.589)	0.406 (0.365, 0.448)	0.375 (0.332, 0.42)
P10	CoT	0.486 (0.393, 0.565)	0.354 (0.315, 0.395)	0.291 (0.248, 0.335)
P11	CoT + CC	0.584 (0.511, 0.64)	0.442 (0.399, 0.485)	0.417 (0.372, 0.463)
P11_1	CoT + CC	0.589 (0.517, 0.644)	0.451 (0.41, 0.496)	0.428 (0.383, 0.473)
P11_2	CoT + CC	0.591 (0.525, 0.644)	0.463 (0.421, 0.506)	0.443 (0.397, 0.487)
P11_3	CoT + CC	0.636 (0.577, 0.685)	0.504 (0.461, 0.545)	0.488 (0.444, 0.534)
P11_4	CoT + CC	0.601 (0.543, 0.644)	0.528 (0.487, 0.571)	0.518 (0.474, 0.564)
P11_5	CoT + CC	0.563 (0.504, 0.607)	0.425 (0.382, 0.466)	0.404 (0.359, 0.45)
P12	2 CoT + CC	0.539 (0.456, 0.604)	0.403 (0.361, 0.444)	0.371 (0.328, 0.418)
P12_1	2 CoT + CC	0.588 (0.506, 0.652)	0.459 (0.418, 0.502)	0.422 (0.375, 0.466)
P12_2	2 CoT + CC	0.605 (0.548, 0.651)	0.464 (0.425, 0.507)	0.445 (0.398, 0.491)

Abbreviations: CI, 95%-confidence interval.

Table S9: All performance metrics per prompting strategy for GPT-3.5-turbo on enriched dataset without keywords. Text in bold indicates the best performance.

Prompt	Concept	Precision (CI)	Recall (CI)	F1-Score (CI)
P1	zero-shot	0.471 (0.373, 0.561)	0.341 (0.301, 0.38)	0.271 (0.23, 0.314)
P2	zero-shot	0.429 (0.305, 0.613)	0.272 (0.236, 0.311)	0.192 (0.158, 0.23)
P3_1	CC	0.57 (0.495, 0.628)	0.442 (0.401, 0.483)	0.411 (0.367, 0.456)
P3_2	CC	0.549 (0.481, 0.606)	0.448 (0.406, 0.491)	0.419 (0.374, 0.464)
P4_1	CC	0.574 (0.51, 0.616)	0.433 (0.391, 0.476)	0.409 (0.364, 0.454)
P4_2	CC	0.558 (0.497, 0.603)	0.431 (0.39, 0.472)	0.412 (0.368, 0.456)
P5	CC	0.625 (0.558, 0.673)	0.507 (0.464, 0.551)	0.488 (0.443, 0.534)
**P6**	**CC**	**0.573 (0.518, 0.615)**	**0.526 (0.483, 0.569)**	**0.518 (0.473, 0.561)**
P7	CoT	0.45 (0.319, 0.575)	0.298 (0.26, 0.337)	0.225 (0.187, 0.265)
P9	CoT + CC	0.54 (0.455, 0.609)	0.399 (0.358, 0.442)	0.365 (0.319, 0.409)
P9_1	CoT + CC	0.559 (0.478, 0.622)	0.423 (0.381, 0.466)	0.395 (0.351, 0.441)
P10	CoT	0.476 (0.364, 0.563)	0.348 (0.309, 0.391)	0.281 (0.24, 0.324)
P11	CoT + CC	0.553 (0.482, 0.608)	0.449 (0.406, 0.493)	0.422 (0.378, 0.468)
P11_1	CoT + CC	0.591 (0.527, 0.642)	0.464 (0.423, 0.507)	0.446 (0.401, 0.492)
P11_2	CoT + CC	0.595 (0.528, 0.647)	0.461 (0.419, 0.502)	0.442 (0.395, 0.486)
P11_3	CoT + CC	0.632 (0.568, 0.677)	0.509 (0.468, 0.554)	0.487 (0.442, 0.533)
P11_4	CoT + CC	0.588 (0.527, 0.629)	0.519 (0.478, 0.562)	0.509 (0.464, 0.552)
P11_5	CoT + CC	0.556 (0.494, 0.602)	0.418 (0.375, 0.459)	0.392 (0.348, 0.439)
P12	2 CoT + CC	0.525 (0.44, 0.597)	0.395 (0.354, 0.438)	0.36 (0.316, 0.405)
P12_1	2 CoT + CC	0.604 (0.517, 0.669)	0.459 (0.418, 0.502)	0.422 (0.376, 0.467)
P12_2	2 CoT + CC	0.6 (0.542, 0.647)	0.453 (0.412, 0.496)	0.437 (0.392, 0.483)

Abbreviations: CI, 95%-confidence interval.

Table S10: Performance metrics of In-context learning-strategy: few-shots for GPT-3.5-turbo.

Technique	Precision	Recall	F1-score
Random few-shots	0.313	0.324	0.314
K-nearest neighbour	0.279	0.198	0.155

Performance is measured on unenriched dataset.
'Random few-shots' is the technique in which the classification examples (few-shots) are picked randomly from the train split. 'K-nearest neighbor' stands for the technique which, as few-shots, uses the closest neighbors from the train split to each test example.
Our approach is based on the following literature [33] and [34].

Figure S2: Radar plot of the per-class F1 performance of the top experiment set-ups with SciBERT, GPT-3.5 and GPT-4.

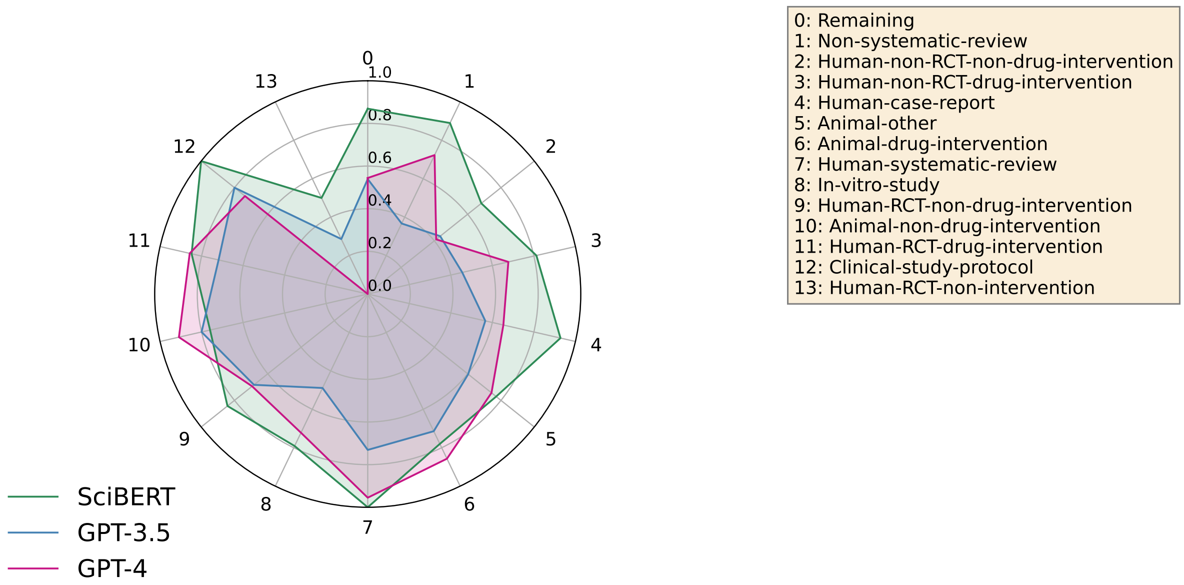

Supplement: Emilova Doneva et al. supplementary material [file S1759287925100318sup001.pdf]
